# Evolution of MAF and OTX families during the emergence and stabilization of rod photoreceptors across Metazoans

**DOI:** 10.21203/rs.3.rs-10144266/v1

**Published:** 2026-09-11

**Authors:** Soumitra Pal, Noor D. White Carreiro, Zachary Batz, Leo Goubet-McCall, Anand Swaroop

**Affiliations:** 1 Neurobiology, Neurodegeneration and Repair Laboratory, National Eye Institute, National Institutes of Health, Bethesda, Maryland, United States of America; 2: Biological Imaging Core, National Eye Institute, National Institutes of Health, Bethesda, Maryland, United States of America

**Keywords:** NRL, CRX, MAF, OTX, photoreceptor, retina, evolution

## Abstract

A central question in evolutionary developmental biology is how gene regulatory networks are rewired to generate new cell types. In mammals, rod specification and maintenance depend on the MAF-family transcription factor NRL; yet, non-mammalian vertebrates also possess NRL-independent rods, suggesting that alternative regulatory mechanisms may underlie rod evolution across lineages. Here, we investigate the evolutionary history and functional diversification of the MAF transcription factor family across metazoans. Phylogenetic and comparative genomic analyses reveal that large and small MAFs expanded through ancient duplication events, whereas the ancestral large and small *MAF* genes predate metazoan diversification. We uncover signatures of episodic positive selection and markedly elevated expression of *NRL* in mammals compared to non-mammalian orthologs. This upregulation is associated with cis-regulatory elements that include binding sites for the OTX-family homeodomain proteins, OTX2 and CRX. Furthermore, we identify evidence of co-evolution between the DNA-binding domain of NRL and its interacting partner CRX, suggesting coordinated evolution of transcription factor complexes together with their target *cis*-regulatory sequences. Our findings support a model in which gene duplication, regulatory innovation, and protein co-evolution collectively drive the emergence and stabilization of mammalian rod photoreceptor identity, with NRL playing a pivotal role in enabling mammalian survival through the Mesozoic era. These studies highlight context-specific deployment of conserved transcription factor networks to generate new features and functions during evolution of sensory systems in vertebrates.

## INTRODUCTION

Cellular diversity in multicellular organisms can be understood through the evolution of regulatory networks that produce and maintain specific gene expression patterns ^1^. Morphological and functional innovations often arise not through the invention of entirely new genes but by changes in spatiotemporal and quantitatively precise expression of regulatory genes ^2-5^. Transcription factors and their cognate *cis*-regulatory elements play a fundamental role in enabling context-specific gene regulation and rewiring of developmental programs ^6^. Such regulatory adaptations are particularly important in the evolution of highly specialized cell types, such as sensory neurons, where precise control of gene expression is essential for determining cell fate and function ^7,8^. Despite growing insights into gene regulatory networks, a major unresolved question remains: how have conserved regulatory components been differentially deployed and modified over evolutionary time to generate new cell identities?

The retina provides a powerful model for addressing evolution of regulatory complexity. The light-sensitive cells, the photoreceptors, initiate vision and their origins can be traced to ancestral light-detecting cells in early bilaterians ^9^. The photoreceptors as well as the retina have diversified extensively across metazoans, incorporating layers of complex information processing systems that reflect the essential role of vision in evolution of species with higher order functions, yet key aspects of their molecular and developmental architecture remain conserved ^10-13^. In vertebrates, the retina exhibits a highly stratified laminar structure containing six major types of neurons, including two primary classes of photoreceptors: cones and rods ^13-15^. Cones mediate high-acuity and color vision under photopic conditions, whereas rods enable dim light vision with enhanced sensitivity but low spatial resolution and no color discrimination ^10,16,17^. Multiple lines of evidence including morphological features and transcriptomic analyses across vertebrate lineages support the hypothesis that cones represent the ancestral photoreceptor type, with rods emerging later through modification and partial co-option of cone gene regulatory networks ^18,19^. This evolutionary transition likely required both the repression of cone-specific programs and the activation of rod-specific transcriptional states ^7,20,21^, highlighting the importance of regulatory innovation in the evolution of new cell identities and functions.

The rod photoreceptor specification in mammals is determined by neural retina leucine zipper (NRL), a basic motif leucine zipper (bZIP) transcription factor of the musculoaponeurotic fibrosarcoma (MAF) family ^22^ and governed by interacting transcription factor networks, prominently involving and orthodenticle homeobox (OTX) families. MAF transcription factors share a conserved bZIP DNA-binding domain and are divided into large (cMAF, MAFA, MAFB and NRL) and small MAF subgroups (MAFK, MAFF and MAFG) based on the presence or absence of a transactivation domain (Fig. 1A) ^23,24^. In parallel, OTX family members—including OTX1, OTX2, and cone-rod homeobox (CRX)—are homeodomain transcription factors that play essential roles in photoreceptor development and maintenance ^25,26^. These factors function cooperatively within a shared regulatory network: OTX2 and CRX activate photoreceptor-specific genes, including NRL, whereas CRX physically interacts with NRL to drive rod-specific transcriptional programs ^27,28^. Together, these interactions define a core transcriptional module that integrates upstream regulatory inputs with downstream gene expression programs to stabilize rod identity ^7,29^.

The large MAF transcription factor NRL acts as the master regulator of rod photoreceptor fate and is both necessary and sufficient for rod specification in the mammalian retina ^22,30^. NRL promotes rod identity by activating rod-specific genes while repressing cone-specific transcriptional programs, and ectopic expression of NRL can reprogram cone precursors toward a rod-like phenotype. Its expression is tightly controlled by upstream regulators, including OTX2 and CRX, which bind conserved cis-regulatory elements to initiate and maintain NRL transcription during photoreceptor differentiation ^31,32^. However, this NRL-centered regulatory architecture operating in mammals is not conserved across all vertebrates. NRL-independent mechanisms of rod development have been reported in birds, which lack *bona fide* NRL, and zebrafish, which utilizes both NRL-dependent and independent pathways ^18,33,34^. In these systems, closely related large MAF paralogs—such as MAFA in birds ^35^ and MAFB (or mafba) in fish ^33,36^ have been implicated in rod specification, suggesting that rod identity can be supported by alternative large MAF factors. Thus, a more flexible ancestral regulatory framework in which MAF paralogs are deployed within a conserved MAF- and OTX-centered network seems to drive rod-like programs during evolution. Several fundamental questions can be raised from an evo-devo perspective. How did MAF and OTX transcription factors, and their interactions, evolve to produce distinct regulatory architectures across vertebrate lineages? How has the broader MAF transcription factor family diversified and been redeployed across vertebrate evolution to support rod developmental programs? And how did NRL become central to rod photoreceptor identity in mammals?

To address how gene (and genome) duplication and regulatory rewiring have shaped the evolution of rod photoreceptor identity, we have investigated the evolutionary history and functional diversification of the MAF and OTX transcription factor families across metazoans. By integrating publicly available annotated protein sequences with *de novo* reconstructions from newly derived transcriptomic data, we report integration of phylogenetic, expression, and sequence analyses to reconstruct the origins and expansion of large and small MAFs and assess lineage-specific patterns of selection. We further examine the co-evolution of MAF proteins with their OTX-family partners and identify cis-regulatory innovations associated with NRL expression. Our results provide insights into conserved regulatory networks that are repurposed to generate novel cell types during vertebrate evolution.

## RESULTS

### Conservation and lineage-specific expansion of MAF and OTX transcription factors across metazoans

To investigate the evolutionary origins of MAF and OTX family proteins, we first traced their history from early animals to placental mammals. This evolutionary trajectory spans major phylogenetic groups, including cnidarians, protostomes, echinoderms, cephalochordates (lancelets), urochordates (tunicates), jawless vertebrates (agnathans including cyclostomes such as lampreys and hagfish), cartilaginous fishes, ray-finned fishes (including teleosts), lobe-finned fishes, amphibians, reptiles (turtles and crocodilians), birds, and mammals. Two rounds of whole-genome duplication (2R-WGD) in an ancestral vertebrate lineage are suggested to be the major driver of vertebrate genomic innovation ^37^, a concept supported by comparative genomic and synteny analyses across vertebrate genomes (Fig. 1B) ^38-43^. These studies indicate an early vertebrate genome duplication that is followed by a second duplication associated with the emergence of jawed vertebrates. On the other hand, cyclostomes (agnathans) exhibit evidence of independent genome duplications or triplications. Subsequent lineage-specific duplications further expanded gene repertoires, including the teleost-specific whole-genome duplication (3R) and additional duplications in certain amphibian lineages such as frogs.

To systematically identify MAF and OTX family across vertebrates and related lineages, we analyzed 181 species spanning Metazoa (Fig. 1C, Table S1). Even though annotated genomes provided prediction of protein sequences, many gene models remained incompletely curated and were often assigned generic identifiers (for example, LOC entries). Additionally, it remained unclear whether the seven MAF transcription factors and three OTX transcription factors that are characteristic of jawed vertebrates were conserved in jawless vertebrates and invertebrate lineages. Our systematic gene identification and curation across all species identified a single large MAF (MAFL), a single small MAF (MAFS), and one OTX family member in invertebrates (Fig. 2A). Jawless vertebrates encoded four large MAFs (MAFL1A, MAFL1B, MAFL2A, MAFL2B), one small MAF (MAFS1A), and four OTX (OTX1C, OTX2A, OTX2B, OTX2C) proteins, whereas jawed vertebrates exhibited the canonical collection of four large MAFs (CMAF, NRL, MAFA MAFB), three small MAFs (MAFF, MAFG, MAFK), and three OTX paralogs (OTX1, OTX2, CRX). Despite this orthology-based framework, annotation gaps persisted, particularly in less-studied jawless vertebrates and invertebrate taxa. We also observed apparent lineage-specific absences or sparse annotations, including the lack of detectable NRL in birds and cartilaginous fishes, limited CRX annotations in avian species, and sparse OTX2 annotations in teleost fishes (Fig. S1).

To address these issues, we leveraged an expanded retinal transcriptomic dataset comprising 219 RNA-seq samples from 107 of the 181 selected species (Table S2). Of these, 61 samples were generated in this study, and the remainder were obtained from Gene Expression Omnibus data repository (https://www.ncbi.nlm.nih.gov/geo/). Using these resources, we implemented a complementary *de novo* protein prediction pipeline to recover previously unannotated or incomplete MAF and OTX sequences. No *de novo* NRL sequence was predicted for birds and cartilaginous fishes, yet CRX representation improved among avian species whereas OTX2 representation improved in teleost fishes (Fig. 2A, S1). Our curation yielded 426 annotated large MAF, 335 annotated small MAF, and 364 annotated OTX sequences, in addition to 381 *de novo* large MAF, 237 *de novo* small MAF, and 321 *de novo* OTX predictions. In total, our analysis uncovered 807 large MAF, 572 small MAF, and 685 OTX sequences across the dataset (Table S3).

To obtain a global overview of MAF and OTX protein evolution across our curated dataset, we generated latent protein embeddings using the ESM-C (Evolutionary Scale Modeling – Cambrian) protein language model ^44^ and visualized their distribution by principal component analysis (PCA). The embeddings robustly separated MAF from OTX proteins and further distinguished large MAFs from small MAFs (Fig. 2B). Proteins clustered consistently by family across species, indicating that the learned representations capture conserved sequence features. Notably, a subset of *de novo* small MAF predictions localized closer to the OTX cluster, likely reflecting the truncation and incomplete sequence recovery described above. We therefore restricted subsequent analyses to the annotated large MAF set. Strikingly, PCA of annotated large MAF proteins revealed a structured evolutionary trajectory: species divergence was reflected along a continuum, whereas paralog-specific trajectories became increasingly distinct over evolutionary time (Fig. 2C). In particular, MAFA and MAFB followed closely aligned paths, but NRL and cMAF diverged along separate directions.

### Vertebrate MAF genes originated through ancient whole genome duplications

#### Phylogeny results

The large MAF phylogeny (Fig. 3A) reveals a clear pattern. In jawed vertebrates, large MAF proteins form four clades—cMAF, NRL, MAFA, and MAFB—organized into two higher-order groups, with cMAF–NRL forming one superclade and MAFA–MAFB forming the other (Fig. 3A, blue nodes). In contrast, jawless vertebrate large MAF–like proteins form four subclades that do not correspond directly to the canonical large MAF proteins. Instead, they cluster into two higher-order groups (Fig. 3A, orange nodes) paralleling the cMAF–NRL and MAFA–MAFB superclades. To avoid implying direct orthology, we designate these orthologs MAFL1A/B and MAFL2A/B. This topology is consistent with an early duplication of a single ancestral large MAF gene (Fig. 3A, yellow node), followed by independent lineage-specific duplications in jawless and jawed vertebrates, which generated their present repertoires.

The phylogeny of small MAF proteins reveals a related but distinct evolutionary pattern (Fig. 3B). Jawed vertebrates contain three small MAF clades: MAFF, MAFG, and MAFK where MAFG and MAFK form a closely related superclade. In contrast, jawless vertebrates retain a single small MAF lineage (MAFS1A), which clusters with the MAFG–MAFK group (Fig. 3B, blue node) rather than with MAFF. These relationships suggest that diversification of a vertebrate-specific ancestral small MAF gene (Fig. 3B, yellow node) occurred primarily within the jawed vertebrate lineage, whereas cyclostomes retained a reduced repertoire.

A combined phylogeny of large and small MAF proteins (Fig. 3C) reveals a distinct separation between large and small MAF families, although deeper branching relationships do not fully mirror species topology. Nevertheless, the tree structure indicates an ancestral duplication early in metazoan evolution, separating the large and small MAF lineages which subsequently diversified independently.

Together, these phylogenetic analyses indicate that the MAF transcription factor family expanded through a combination of ancient duplications establishing the large and small MAF families, followed by lineage-specific duplications that shaped the repertoires of jawless and jawed vertebrates.

#### Synteny supports whole-genome duplication origins

To test whether these duplications reflect whole-genome duplication (WGD) events, we examined the syntenic context of MAF genes across representative vertebrates (Fig. S2-S4), including six jawed vertebrates (human, mouse, opossum, western clawed frog, chicken, and spotted gar), two cyclostomes (sea lamprey and brown hagfish), and the invertebrate chordate outgroup amphioxus. For large MAF genes (Fig. S2), the conserved genomic neighborhoods support a shared ancestral chromosomal context. Paralogous gene families—*NDRGx*, *CHDx*, *TOXy*, *JPHx*, *CPNEx*, and *ADCYx*—consistently flank each of the four large MAF loci in jawed vertebrates and the single large MAF locus in amphioxus. Interestingly, all these flanking genes as well as *NRL* are absent in chicken. In cyclostomes, although phylogenetic analysis identified only four large MAF genes (*MAFL1A*/*B* and *MAFL2A*/*B*), synteny analysis reveals six chromosomal segments containing paralogous copies of these neighboring genes. These segments correspond to chordate linkage groups (CLGs)—a consensus set of chromosome (or chromosomal arms) for ancestral chordates derived from chromosome-scale genome sequencing of amphioxus, lamprey and invertebrates ^40,45^. In particular, the segments belong to CLG-D ^43^, consistent with cyclostome-specific genome triplication events. Thus, the cyclostome lineage likely possessed six ancestral large MAF paralogs (MAFL1A/B/C and MAFL2A/B/C), and two of these are subsequently lost during evolution.

A comparable synteny analysis of small MAF genes (Fig. S3) reveals that paralogous gene families—*SYNGRx*, *RBFOXy*, *ANKRDx*, *CARDx*, *CYTHx*, and *RACx*—consistently flank the three small MAF loci in jawed vertebrates. These chromosomal regions correspond to chordate linkage group H (CLG-H) ^43^, indicating that small MAF genes also originated through ancient WGD events. Consistent with cyclostome genome architecture, these patterns suggest that the cyclostome ancestor likely possessed six small MAF paralogs (MAFS1A/B/C and MAFS2A/B/C), of which only a single lineage is retained following subsequent gene loss.

#### OTX genes show parallel duplication history

The whole-genome origin of the OTX gene family has been recognized previously ^26^, where OTX1 was shown to diverge during the first round of vertebrate genome duplication and the second duplication produced OTX2 and CRX. However, the earlier analyses did not account for cyclostome-specific duplications. To address this, we performed a parallel phylogenetic and synteny analysis of OTX genes (Fig. S4). These analyses show that OTX loci belong to chordate linkage group A1 (CLG-A1), as indicated ^43^. Our results suggest that cyclostomes originally possessed six OTX paralogs; of these four – OTX1C and OTX2A/B/C – are retained, whereas the other two are lost subsequently.

### Mammalian NRL experienced episodic positive selection

To examine the evolutionary dynamics of NRL, we conducted a selection analysis to identify specific lineages or subtrees that have undergone episodic diversifying selection. The subtree showing the strongest evidence of positive selection is the mammalian NRL lineage (Fig. S5), suggesting that NRL in mammals has undergone adaptive changes not observed in other vertebrate groups. The absence of such a signal in non-mammalian lineages points to a specific evolutionary trajectory, potentially reflecting unique visual or physiological demands in mammalian habitats. Episodic selection acting on mammalian NRL may have contributed to fine-tuning of photoreceptor development and function in response to ecological pressures. These results highlight the importance of NRL as the target of adaptive innovation in mammals and raise new questions about how specific amino acid changes in this lineage shaped the evolution of mammalian phototransduction.

### NRL expression is high in mammals

Across invertebrates, large *MAF* genes are expressed at low to moderate levels (0–5 log CPM; Fig. 4). In jawless vertebrates, expression is highly restricted, with only *MAFL1B* showing detectable expression (~4 log CPM). In jawed vertebrates, *cMAF* exhibits consistently low expression across species. In contrast, the remaining large *MAF* genes exhibit lineage-specific expression patterns. In cartilaginous and non-teleost fishes, *MAFB* is expressed at moderate to high levels. In teleost fishes and amphibians, both *NRL* and *MAFB* show moderate to high expression. In squamates, only *MAFB* reaches moderate levels, whereas in turtles, both *NRL* and *MAFB* are expressed at moderate to high levels. In birds, *MAFA* is the predominant medium to highly expressed paralog, whereas in mammals, *NRL* exhibits the highest expression levels (~8 log CPM). In all other lineage–gene combinations, expression remains low.

We further analyzed single cell RNA-seq data ^46^ from 16 jawed vertebrates and sea lamprey to examine large MAF expression at the cell-type level (Fig. 4). In rod photoreceptors, MAF expression patterns recapitulate those observed in bulk RNA-seq data, with *NRL* showing markedly elevated expression in mammalian rods. Notably, in the original lamprey single cell study ^47^, rod-like and cone-like photoreceptors are annotated as PR1 and PR2, respectively, and the gene labeled as *MAF2* corresponds to *MAFL1B* in our annotation.

Together, these results indicate that, in addition to sequence diversification, *MAF* genes have undergone lineage-specific shifts in expression, consistent with functional specialization at the transcriptional level, with *NRL* showing pronounced upregulation in mammals.

### Mammal-specific regulatory elements drive elevated *NRL* expression

To investigate the mechanism underlying very high *NRL* expression in mammals, we examined the expression of its known upstream regulators, OTX2 and CRX, across vertebrates. Surprisingly, comparative expression analysis shows stable expression levels of *OTX2* or *CRX* transcripts in vertebrates with no apparent mammal specific increase (Fig. 4). Thus, augmented *NRL* expression in mammals cannot be explained by increased expression of its canonical regulators, prompting us to investigate whether mammal-specific regulatory elements at the *NRL* locus might account for this discrepancy ^48^. Prior comparative genomic analysis identified three conserved regulatory regions upstream of *NRL* that represent evolutionary innovations within mammals, with one (region “B”) showing little or no conservation in non-mammalian vertebrates ^31,32,49^ (Fig. S6A-B; UCSC Genome Browser hg38). Region B was later shown to be sufficient to drive *Nrl* promoter activity in mouse photoreceptors ^32^ (Fig. S6C). The restricted presence of this enhancer in mammals strongly suggests its contribution to the evolutionary increase in *NRL* expression. The emergence of this regulatory element therefore likely represents a key innovation that enhanced NRL activity in the mammalian retina. We however can’t exclude the role of other potential upstream regulators of NRL expression at this stage.

### DNA-binding domain of the mammalian NRL shows co-evolution with the CRX homeodomain

OTX2 and CRX can also physically interact with NRL to drive rod-specific gene expression ^27,50^, prompting us to evaluate whether their DNA-binding domains co-evolved across vertebrates. Large MAF proteins, including NRL, contain a transactivation domain as well as a DNA-binding region, which encompasses the extended homology region (EHR), a basic region, and leucine zipper (see Fig. 1A). In contrast, OTX proteins possess a homeodomain that mediates DNA binding in addition to a C-terminal OTX domain. To test for co-evolution between these families, we computed pairwise Pearson correlations (PCC) of sequence similarity scores between each OTX domain and each MAF domain (Fig. 5, S7). This approach allowed us to assess whether evolutionary changes in one domain are mirrored by correlated changes in another across vertebrate species. The resulting heatmaps reveal a striking pattern of within-mammals similarity to the exclusion of other vertebrate groups (Fig. 5A). Furthermore, the CRX homeodomain shows a strong correlation with the NRL EHR+basic region, whereas OTX2 displays little to no correlation with any NRL domains (Fig. 5B, left). This correlation is based on all vertebrate species but becomes especially pronounced when the analysis is restricted to mammals (Fig. 5B, middle). In contrast, the correlation disappears entirely in non-mammalian vertebrates (Fig. 5B, right), suggesting that the co-evolutionary relationship between CRX and NRL domains is a mammal-specific innovation. Functionally, this aligns with the known cooperative role of CRX and NRL in activating rod-specific gene networks, including RHO (rhodopsin) and other phototransduction genes ^27,51^. The emergence of domain-level co-evolution may have fine-tuned the physical and functional partnership between CRX and NRL, reinforcing rod specialization in mammals. These findings highlight not only regulatory and expression-level adaptations of NRL but also structural innovations at the interaction interface that may have been essential for evolution of vision in mammals.

### An integrated model for MAF family evolution: Large and small MAF families diverged early in animal evolution

Having established that NRL underwent mammal-specific changes in expression, regulatory control, protein sequence evolution, and domain-level co-evolution with CRX, we next integrated these findings with our broader phylogenetic and synteny analyses to reconstruct the deeper evolutionary history of the MAF family. This synthesis places the mammalian specialization of NRL within a longer evolutionary framework, beginning with the origin of distinct large and small MAF lineages and followed by vertebrate genome duplications, cyclostome-specific expansions, and lineage-specific gene loss.

Large and small MAF proteins form two well-separated clades, indicating an early divergence of these families (see Fig. 3C); however, synteny analysis does not support a recent whole-genome duplication (WGD) origin for this split (Figs. S2-S3). In amphioxus which approximates a pre-vertebrate genomic state, the genes flanking the putative large and small MAF loci on chromosomes 7 and 8, respectively, are not paralogous, indicating that these loci do not derive from duplicated chromosomal segments in this lineage. Consistent with this, in vertebrates, large and small MAF genes map to distinct chordate linkage groups (CLG-D and CLG-H, respectively), suggesting that they originated from separate ancestral chromosomal regions rather than from a single WGD event. Based on these observations, we propose a model of MAF gene evolution (Fig. 6A). The ancestral bilaterian genome likely contained a single MAF-like gene, which subsequently gave rise to ancestral large and small MAF lineages through an early duplication event predating metazoa evolution as CLGs are based on metazoan genomes ^40,45^. The absence of shared flanking paralogs in amphioxus, together with their localization to distinct chromosomal scaffolds, argues for a tandem or small-scale duplication origin event predating metazoans that separated the two loci.

Subsequent vertebrate whole-genome duplications (2R-WGD) expanded these ancestral lineages, giving rise to the four large MAF genes (*cMAF*, *NRL*, *MAFA*, *MAFB*) and three small MAF genes (*MAFF*, *MAFG*, *MAFK*) observed in jawed vertebrates (Fig. 6A). In cyclostomes, additional lineage-specific genome duplications or triplications further expanded these loci, followed by gene loss, resulting in the reduced complements observed today (four large MAFs: *MAFL1A*/*B* and *MAFL2A*/*B*; one small MAF: *MAFS1A*).

Although it remains unclear whether ancestral large and small MAF loci were physically linked prior to vertebrate duplications, partial synteny conservation in extant genomes suggests possible ancestral associations. For example, in frog, *cMAF* and *MAFF* (chromosome 4) and *MAFB* and *MAFG* (chromosome 10) reside in related genomic contexts, suggesting tentative linkage relationships, whereas *MAFA* and *MAFK* form a third pair. These associations (indicated schematically by dotted lines in Fig. S2) remain speculative and require further validation.

Overall, phylogenetic and synteny analyses support a model in which large and small MAF families diverged early, before vertebrate genome duplications, and were subsequently expanded through vertebrate WGD events and shaped by lineage-specific gene retention and loss. Within this broader framework, mammalian NRL represents a later specialization of one large MAF paralog, characterized by elevated retinal expression, mammal-specific regulatory innovation, episodic positive selection, and co-evolution with the CRX homeobox domain.

## DISCUSSION

### Stepwise assembly of the MAF regulatory framework in vertebrate photoreceptors

By combining curated annotations with *de novo* predicted sequences across a broad phylogenetic range, we refine the evolutionary history of the MAF and OTX transcription factor families. The identification of homologs in both invertebrates and early-diverging vertebrates extend previous models and provide a more resolved view of the origins of this regulatory network. Large MAF and OTX genes are expressed at low to moderate levels in invertebrates, consistent with the absence of a rod-specific transcriptional program ^8,13,52^. Following two rounds of vertebrate whole-genome duplication (2R-WGD), ancestral MAF genes appear to have expanded into multiple paralogs (ohnologs), providing the basis for functional diversification ^37,38,53^. Our phylogenetic and syntenic analyses clarify the relationships among large and small MAF subfamilies and reveal both shared and lineage-specific duplication histories in jawless and jawed vertebrates. From an evo-devo perspective, these duplications established a modular and expandable transcription factor toolkit that could be differentially deployed to generate distinct photoreceptor identities.

### Ancestral and non-mammalian rod programs: functional flexibility of large MAFs

Our analyses suggest that vertebrates likely employed a flexible, MAF-dependent mechanism for rod-like photoreceptor specification and maintenance, rather than a strictly NRL-dependent program. In jawless vertebrates, MAFL1B appears to fulfill this role in rod-like photoreceptors of the primitive duplex retina, as both bulk and single cell RNA-seq data show above-moderate expression of this paralog.

Among jawed vertebrates, regulatory strategies vary across lineages. Cartilaginous and non-teleost fishes lack NRL, with MAFB likely serving an analogous function ^54^. In teleost fishes, both NRL and MAFB orthologs are moderately expressed, consistent with the presence of both *nrl*-dependent and *mafba*-dependent rod subtypes ^36^. In reptiles, birds lack NRL and instead rely on MAFA ^18^, whereas other lineages retain NRL for rod maintenance. Across these diverse systems, a common feature emerges where rod identity is consistently associated with moderate to high expression of a large MAF paralog, rather than a single conserved gene.

In addition to expression-level variation, structural differences among large MAF proteins may contribute to functional specialization. NRL lacks specific regions present in other large MAFs, and similar truncations are observed in bird MAFA and teleost mafba. This pattern suggests that rod-promoting activity may depend on shared structural configurations rather than strict orthology, highlighting a potential case of functional convergence within a conserved transcription factor family. Experimental validation will be required to test this hypothesis.

### Emergence of a specialized NRL-centered rod program in mammals

In contrast to the flexible ancestral state, our data support a transition toward a specialized, NRL-centered regulatory program in mammals, marked by coordinated changes at multiple levels. We detect signatures of episodic positive selection in mammalian NRL, indicating lineage-specific protein evolution, alongside substantially elevated *NRL* expression relative to other vertebrates. In addition, mammal-specific cis-regulatory elements exist upstream of *NRL*, including a conserved enhancer (region B) enriched for CRX/OTX2 binding, likely contributing to increased transcriptional activity.

Based on the findings reported here, we propose a model in which mammalian NRL was optimized through both coding and regulatory evolution. This multi-layered adaptation aligns with evo-devo principles in which developmental regulators are modified through combined sequence and cis-regulatory changes to generate lineage-specific traits ^5,48,55^. Such regulatory stabilization may have contributed to the emergence of rod-dominant retinas in mammals, and thus the evolutionary success and radiation of the mammalian species. During the Mesozoic era, or “age of dinosaurs,” the ability of mammals to adapt to low-light environments and become nocturnal (the evolutionary “nocturnal bottleneck”) allowed them to escape predation and survive as a lineage ^18^. After the mass extinction event at the end of the Cretaceous period, the release of the predation pressure imposed by dinosaurs allowed mammals to diversify into the radiation that exists today. Adaptation of other senses and the ability of the brain to integrate larger sensory input are also necessary, but we argue that vision is probably the most important of these, and the signature of a rod-dominant retina is still a hallmark of mammalian retinas today. Of course, establishing direct causal links between molecular innovations and phenotypic outcomes would require targeted functional studies.

### Co-evolution of transcription factors and regulatory networks

Beyond individual genes, our results highlight the importance of coordinated evolution within gene regulatory networks. We observe evidence of mammal-specific co-evolution between the CRX homeodomain and the NRL bZIP DNA-binding region, suggesting refinement of their cooperative interaction. Given the established synergistic role of the NRL–CRX complex in activating rod-specific transcriptional programs ^27,56^, these findings point to the evolution of transcription factor partnerships as a key mechanism underlying regulatory innovation. More broadly, our analyses suggest that rod photoreceptor evolution reflects not only gene duplication and divergence but also the progressive tuning of interacting regulatory components. This network-level perspective aligns with emerging views in evo-devo that emphasize the evolution of interactions—rather than individual genes—as a primary driver of developmental novelty ^48,57^.

Taken together, our results support a stepwise evolutionary model in which (i) ancestral MAF genes diversified through genome duplication, (ii) early vertebrates employed flexible large MAF–dependent photoreceptor programs, and (iii) mammals evolved a specialized, NRL-centered regulatory architecture associated with increased rod abundance (Fig. 6C). This trajectory illustrates how conserved transcription factor families can be repeatedly redeployed and rewired to meet lineage-specific ecological demands, such as adaptation to dim-light environments. Finally, the evolutionary history of NRL has direct biomedical relevance. Mutations in *NRL* cause diverse retinopathies. The mammal-specific regulatory and structural features identified here may influence the stability and sensitivity of this transcriptional network. Thus, linking evolutionary changes to disease-associated mechanisms provides a framework for understanding how deeply conserved developmental systems become vulnerable to perturbation.

## MATERIALS AND METHODS

### RNA-seq Dataset Generation

A total of 219 bulk RNA-seq samples from retina/eye tissues representing 107 species were included in this study; of these, previously published bulk RNA-seq data from 158 retina samples, representing 86 species, were downloaded from GEO (https://www.ncbi.nlm.nih.gov/geo/), and RNA-seq data from 61 samples (25 species) were generated in house (details are provided in below and in Table S1). In addition, we obtained retinal single cell RNA-seq data from 17 species described in reference ^46^.

For the RNA-seq data generated in this report, retinas were either from commercially purchased tissues or animals, from animals euthanized for other research projects, or from a local raptor rehabilitation center. Commercially purchased animals were euthanized according to National Eye Institute (NEI) Animal Study Protocols 682, 683 and 686 (Branchiostoma floridae, Gymnothorax saxicola, Lepomis macrochirus, Petromyzon marinus, Anolis carolinensis). The Trochilus ^58^ and Columbia livia samples were obtained from colleagues at the National Museum of Natural History. Raptor samples (Accipiter cooperii, Bubo virginianus, Buteo jamaicensis, Megascops asio, Strix varia, Tyto alba) were obtained from Owl Moon Raptor Center where they were euthanized for humane reasons. Colleagues at the University of Maryland provided Alligator mississippiensis, Coturnix japonica, Melopsittacus undulatus, Plethodon cinereus, Serinus canaria and Taeniopygia guttata samples. Colleagues at the NIH provided the Psammomys obesus and Xenopus tropicalis samples, and a colleague at New York University provided the Ciona intestinalis samples. RNA was extracted from whole retinas except in two instances—for lancelets, whole heads were used for RNA extraction, and many whole individuals were used for the microscopic larval tunicates. Tissues were either extracted directly into TRIzol (ThermoFisher Scientific) for immediate RNA extraction or preserved in RNAlater (ThermoFisher Scientific) for 24-48 hr at 4°C and storage at −80°C. All RNA extractions were conducted with TRIzol and a standard phenol:chloroform extraction protocol including two washes with chloroform and two rounds of precipitation with isopropanol. Extracted RNA was resuspended in Ambion RNA storage solution (ThermoFisher Scientific) with the addition of SUPERase-In RNAase inhibitor (ThermoFisher Scientific). RNA quality was assessed on a TapeStation (Agilent), and samples with a RIN above 7 were kept for cDNA synthesis using the Illumina TruSeq RNA kit (Illumina) followed by library preparation via the KAPA library HyperPrep kit (protocol v6.17; Roche). Libraries were amplified 15X, cleaned via SPRI beads to remove adapter dimer, pooled equimolar and sequenced on an Illumina HiSeq 2500.

For each species, RNA-seq reads from all samples were trimmed using trimmomatic v0.39 ^59^, then combined and assembled into a single file to generate a comprehensive species-specific transcriptome using Trinity v2.14.0 ^60^. Coding regions within assembled transcripts were predicted using TransDecoder v5.5.0 ^61^. Transcript abundance was quantified by pseudo-aligning the original reads to the assembled transcriptome using Kallisto v0.48.0 ^62^, with default parameters for paired-end reads and [--single --single-overhang -l 200 -s 20] for single-end reads. Transcripts with within-sample TPM < 1 were discarded. From the remaining set, the longest isoform per putative gene (as defined by Trinity gene clustering) was retained for downstream analyses, resulting in 107 *de novo* species-specific datasets.

### Curation and annotation of MAF and OTX proteins

To annotate MAF and OTX proteins in our *de novo* datasets, we first downloaded available annotations from NCBI, Ensembl or individual publications ^43,63-65^. We then also downloaded published MAF and OTX sequences from an additional 74 metazoan species with available genomic annotations (see ProteinSource = Annotated in Table S2). Species were grouped into invertebrates, jawless vertebrates, and jawed vertebrates, and Orthofinder v3.1.0 ^66^ was run separately on each group to identify orthogroups. Within each orthogroup, proteins with annotated gene symbols corresponding to MAF or OTX were used to assign identities to the remaining members. In cases of conflicting gene symbols, the majority annotation was adopted. For teleost genes with duplicated copies (e.g., a and b paralogs) and for African clawed frog genes with L and S copies, the duplicates were consolidated and treated as a single gene. For orthogroups lacking annotated gene symbols, we assigned identities based on InterProScan v5.72-103.0 ^67^ domain signatures. Specifically, MAF proteins were identified using Conserved Domain Database signatures (cd14697 for general MAF, cd14717 for small MAF, cd14718 for large MAF), FunFam (G3DSA:1.20.5.170:FF:000016), PANTHER (PTHR10129), and Pfam (PF03131, PF08383). OTX proteins were identified using PRINTS (PR01255), with OTX1 further supported by Pfam PF03529 and PRINTS PR01256, and OTX2 by PRINTS PR01257. The resulting reference dataset includes 122 species, 5 genes and 1,123 unique protein sequences (Table S2).

Each of the 107 species-specific *de novo* datasets were queried against a database constructed from the annotated MAF and OTX protein sets described above using BLAST+ v2.5.0 ^68^. Hits with an e-value ≤ 1e-5 and ≥40% sequence similarity were retained as candidates. Candidate sequences were further filtered by requiring the presence of the InterProScan signatures described above for MAF and OTX proteins. Sequence duplicates were removed, and in cases where multiple non-identical candidates remained, a single representative was manually selected based on protein length and sequence similarity to known MAF or OTX proteins. To exclude aberrant predictions, proteins were filtered by length: large MAFs (150–600 amino acids), small MAFs (130–300 amino acids), and OTX proteins (115–500 amino acids). The final set of *de novo* MAF and OTX proteins was merged with the reference dataset and is provided in Table S2. It contains 1,930 unique proteins representing 22 genes and 176 species.

For both annotated and *de novo* predictions, we retained canonical isoforms when specified or otherwise selected the longest predicted isoform. However, several *de novo* predictions likely represent alternative isoforms assigned to distinct gene models, and others were truncated due to insufficient read coverage across full genomic loci. Given these uncertainties and the need for careful isoform resolution, we use the full dataset for downstream expression analyses, but just the curated annotated dataset for phylogenetics and sequence evolution analysis.

Evolutionary Scale Modeling Cambrian (ESMC) embeddings for these proteins were generated using the 300 million–parameter model with the sample code available at https://github.com/evolutionaryscale/esm. Embeddings were extracted from layer 20 for Fig. 2B and layer 29 for Fig. 2C.

### Construction of protein trees

Given that the initial dataset of 181 species was enriched for mammals and birds, we selected 86 representative species to achieve more balanced phylogenetic sampling. Protein alignments were generated by taking into account the three broad groups of metazoans in our dataset (invertebrates, jawless and jawed vertebrates), and using jawed vertebrates as our reference. We first aligned jawed vertebrate sequences using Clustal Omega v1.2.4 ^69^. This alignment was then used to build a profile hidden Markov model (HMM) using HMMER v3.4 ^70^. The proteins from the remaining two groups were then aligned to this profile using hmmalign and trimmed during alignment to remove poorly matching regions. Positions with excessive gaps were filtered using esl-alimask with a gap threshold of 0.5, removing columns where more than 50% of sequences contained gaps. Finally, the original jawed vertebrate sequences and the filtered, HMM-aligned sequences were combined into a single dataset, and a second round of alignment was conducted using Clustal Omega.

For each gene group (small MAFs, large MAFs and OTXs), phylogenetic trees were inferred using FastTree v2.1.11 ^71^. To assess the robustness of the inferred topologies, we generated alternative constrained trees representing different possible pairing relationships among paralogs. For example, among the four large MAFs (e.g., ((NRL, MAFA), (cMAF, MAFB))) and the three small MAFs (e.g., (MAFG, (MAFF, MAFK))) and so on. We computed maximum likelihood scores for each alternative topology and performed topology tests using IQ-TREE’s -z option (v2.3.5) ^72^. This approach implements multiple statistical tests, including the Bootstrap Proportion test using the RELL method (bp-RELL) ^73^, the Expected Likelihood Weight test (c-ELW) ^74^, and the Approximately Unbiased test (p-AU) ^75^. The topology with the highest log-likelihood and the strongest overall statistical support across these tests was selected as the most likely duplication pattern. Large MAF–like proteins from selected non-chordate species were included as outgroups.

The species topology shown in Fig. 1C (without branch lengths) was constructed using information from the NCBI Taxonomy database and TimeTree (timetree.org). Because the resulting tree was non-binary, it was manually resolved into a fully bifurcating topology using phylogenetic relationships reported in the literature ^76-78^. The species tree, gene trees for large MAFs, small MAFs, all MAFs, and all OTXs, together with the corresponding protein alignments, are provided in Supplementary Data File S1.

### Evolutionary analyses

#### Synteny analysis

To assess whether MAF gene expansions are consistent with whole-genome duplication (WGD), we examined conservation of neighboring gene families across species. Rather than relying solely on direct synteny blocks, we used an orthology-guided approach to identify genes located near MAF loci that are orthologous across species for a given MAF gene, but paralogous across different MAF loci—an expected signature of WGD. The analysis was restricted to a representative set of species with high-quality genome annotations: six jawed vertebrates (human, opossum, mouse, chicken, western clawed frog, and spotted gar), two jawless vertebrates (sea lamprey and brown hagfish), and one invertebrate chordate (amphioxus). Scientific names and genome assemblies are listed in Table S1. For each jawed vertebrate genome, genomic regions surrounding each MAF gene were scanned to identify neighboring genes. Candidate paralogous families were defined based on shared gene names (case-insensitive) across species and consistent naming patterns across MAF loci (e.g., RBFOX1, RBFOX2, RBFOX3), indicative of duplication from a common ancestral locus. This heuristic approach enabled identification of multiple conserved paralogous gene families consistent with the 2R-WGD model. Because many flanking genes are incompletely annotated in jawless vertebrates and invertebrates, we ran Orthofinder across all nine species to identify orthologous and paralogous relationships for MAF-associated genomic regions.

To identify regulatory elements upstream of *NRL*, we leveraged our previous work in mouse ^32^, which identified three conserved clusters (A, B, and C) upstream of NRL using the UCSC mm9 (July 2007) 30-species Multiz alignment, with cluster B containing the OTX2/CRX binding sites. From this alignment, which included human, we determined the corresponding human sequences and mapped them to the hg38 (Dec. 2013) genome, visualizing the region using the UCSC 100-vertebrate comparative genomics track (Fig. S6).

#### Sequence evolution analysis

To investigate signatures of sequence evolution, we analyzed vertebrate gene families of interest using HyPhy ^79^. In particular, we applied the adaptive Branch-Site Random Effects Likelihood (aBSREL) method ^80^, which tests for episodic diversifying selection along individual branches of a phylogeny. Because HyPhy requires codon alignments as input, we first retrieved coding DNA sequences for each gene from NCBI. Protein alignments used for constructing phylogenetic trees were converted to codon alignments using the pal2nal utility (Suyama et al., 2006; v14). The resulting codon alignments and corresponding trees were supplied as input to aBSREL, also with default settings. This approach was run in exploratory mode, allowing the algorithm to test all subtrees, including those represented by a single terminal branch. Analysis outputs were visualized using the HyPhy aBSREL web application (http://vision.hyphy.org/aBSREL).

#### Inter-family sequence co-evolution analysis

To assess sequence co-evolution between domains of the OTX and large MAF protein families, we employed a Pearson correlation–based metric following the approach described in reference ^81^. Briefly, for each protein in a family, and each domain within, we computed pairwise amino acid sequence similarities among all species. This yielded a similarity matrix for each OTX domain (OTX1, OTX2, CRX; homeobox and OTX domains) and, analogously, for each large MAF domain (MAFA, MAFB, c-MAF, NRL; transactivation, EHR, basic, bZIP, and combined domains). Each matrix was then flattened into a vector, and the Pearson correlation coefficient (PCC) was calculated between vectors for an OTX domain and a MAF domain. The resulting PCC value served as the co-evolution score for the given domain pair and the corresponding p-value for statistical significance. This procedure was repeated exhaustively across all OTX domains and all MAF domains. Co-evolution scores were visualized as heatmaps, with PCC values shown as text and statistical significance indicated by color (Fig. 5, S7). Analyses were performed across the full vertebrate dataset, as well as separately for mammalian and non-mammalian species groups.

#### Expression evolution analysis

To investigate the evolution of MAF gene expression across Metazoa, we analyzed RNA-seq data from 219 samples spanning 107 species, previously used for *de novo* protein detection (Table S2). For expression quantification, we used annotated surrogate species from the same genus when transcriptome annotations were unavailable for the original species. Trimmed FASTQ files were pseudo-aligned (using Kallisto v0.48.0 ^62^, as above) to the annotated transcriptome of the same species, or a species from the same genus (details in Table S3). Transcript-level Kallisto abundances were summarized at the gene level using the tximport v1.22.0 R package ^82^ with the parameter countsFromAbundance = ‘lengthScaledTPM’.

Because cross-species normalization requires sets of orthologous genes, we partitioned the reference annotations into three groups: invertebrates, jawless and jawed vertebrates. Reference annotations included surrogate annotations when the original species annotation was unavailable; in some cases, a single surrogate reference was used for multiple species. Orthofinder v3.1.0 ^66^ was run separately for each group. Unlike the MAF and OTX curation step, here Orthofinder was restricted to reference species associated with at least one bulk RNA-seq sample. For invertebrates, we retained orthogroups present in at least six (of eight) species and containing at most one paralog per reference species. An additional three orthogroups corresponding to large MAF (*MAFL*), small MAF (*MAFS*), and *OTX*, were manually curated based on sequence evolution analyses, resulting in a total of 3,400 orthogroups. For jawless vertebrates, we retained orthogroups present in at least two (of three) species and containing at most one paralog per reference species. An additional nine orthogroups, four large MAFs (*MAFL1A*/*B*, *MAFL2A*/*B*), one small MAF (*MAFS1A*), and four OTXs (*OTX1C*, *OTX2A*/*B*/*C*), were manually curated based on sequence evolution analyses, yielding 9,909 orthogroups. For jawed vertebrates, orthogroups were retained if they contained one or two genes from species with known paralog expansions (teleosts and Arican clawed frog). We further restricted the analysis to orthogroups containing a single human gene. In addition, ten orthogroups, four large MAFs (*cMAF*, *NRL*, *MAFA*, *MAFB*), three small MAFs (*MAFF*, *MAFG*, *MAFK*), and three OTXs (*OTX1*, *OTX2*, *CRX*), were manually curated based on sequence evolution analyses, resulting in a total of 4,560 orthogroups. The complete orthogroup tables for all three categories are provided in Table S4. In total, 3,400 orthologs in invertebrates, 9,909 in jawless vertebrates, and 4,560 in jawed vertebrates were used for normalization and downstream analyses.

Gene-level abundances for samples within each species group were normalized using the trimmed mean of M-values (TMM) method implemented in edgeR v3.36.0 ^83^, based on the corresponding orthogroup tables. Counts per million were then calculated from the TMM-normalized values using the cpm(log = FALSE) function in edgeR. Normalized expression values for orthogroups in all three categories are provided in Table S5.

#### Pseudobulk analyses of single cell data

For species from reference ^46^, Seurat v5.0.2 ^84^ objects were generated for each of the 17 species except sea lamprey using the corresponding GEO datasets and the analysis scripts provided in the authors’ GitHub repository (https://github.com/shekharlab/RetinaEvolution). For sea lamprey FASTQ files from reference ^47^ were aligned to the reference genome (UKy_Petmar_22M1.pri1.0) using Cell Ranger v10.0.0, retaining only reads mapping to protein-coding genes. For one gene of interest, *LOC116944233* (*MAFL1B*), many reads mapped just outside the annotated gene boundaries, suggesting that the RefSeq annotation was incomplete. In contrast, the gene model reported by reference ^47^ encompassed these reads. We therefore manually updated the annotation to match with ^47^, extending the transcript by 4,481 bp.

For species from reference ^85^, we followed the pipeline described at https://mgi-tech-bioinformatics.github.io/DNBelab_C_Series_HT_scRNA-analysis-software/Document/doc/pipeline/scRNA_en.html to generate Seurat objects. However, with this approach, we were not able reproduce rod photoreceptor clusters from the novel species in that dataset. For each of the 17 species from reference ^46^, pseudobulk expression profiles were generated for genes of interest (MAF and OTX orthologs) across major cell types using the Seurat AverageExpression function. These values were subsequently normalized to a [0–1] scale by dividing each gene’s average expression by its maximum value across cell types.

## Supplementary Material

This is a list of supplementary files associated with this preprint. Click to download.

- PalWhiteNCSuppMaterialFinal06172026.pdf
- PalWhiteNCSuppMaterialFinal07202026.pdf
- PalWhiteNCSuppDataFinal07202026.zip
- PalWhiteNCTableS608052026SP22.xlsx

## Figures and Tables

**Figure 1.**
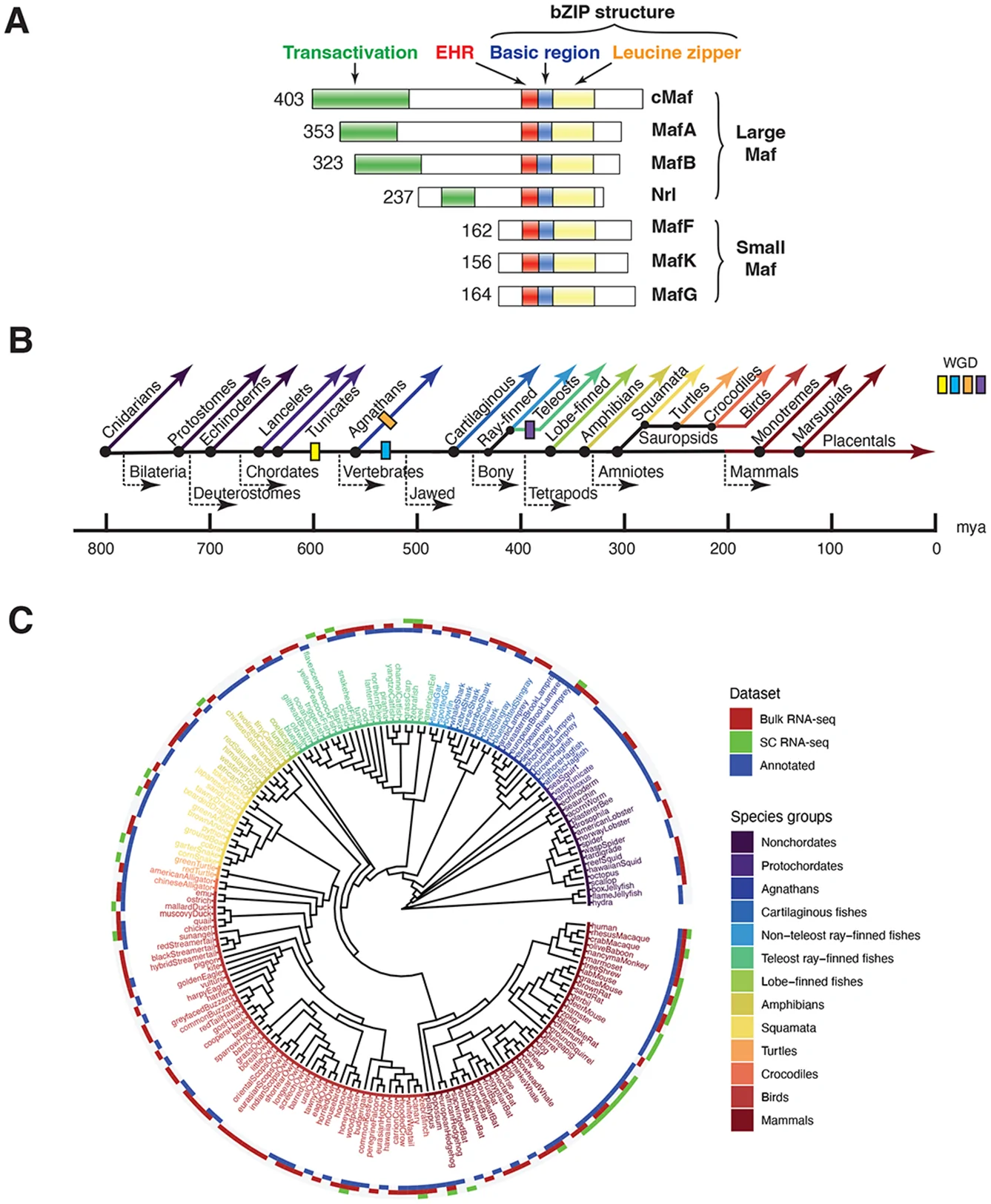
Overview of MAF family proteins, Metaozoan evolution and our dataset. (A) Schematic of the seven MAF proteins in mouse. (B) Timeline tracing the emergence of modern mammals from early metazoan ancestors, indicating major whole-genome duplication (WGD) events. (C) Phylogenetic tree of 181 representative metazoan species used in this study. RNA-seq data availability (single-cell and bulk) and whether the species have genomic and transcriptomic annotations are indicated.

**Figure 2.**
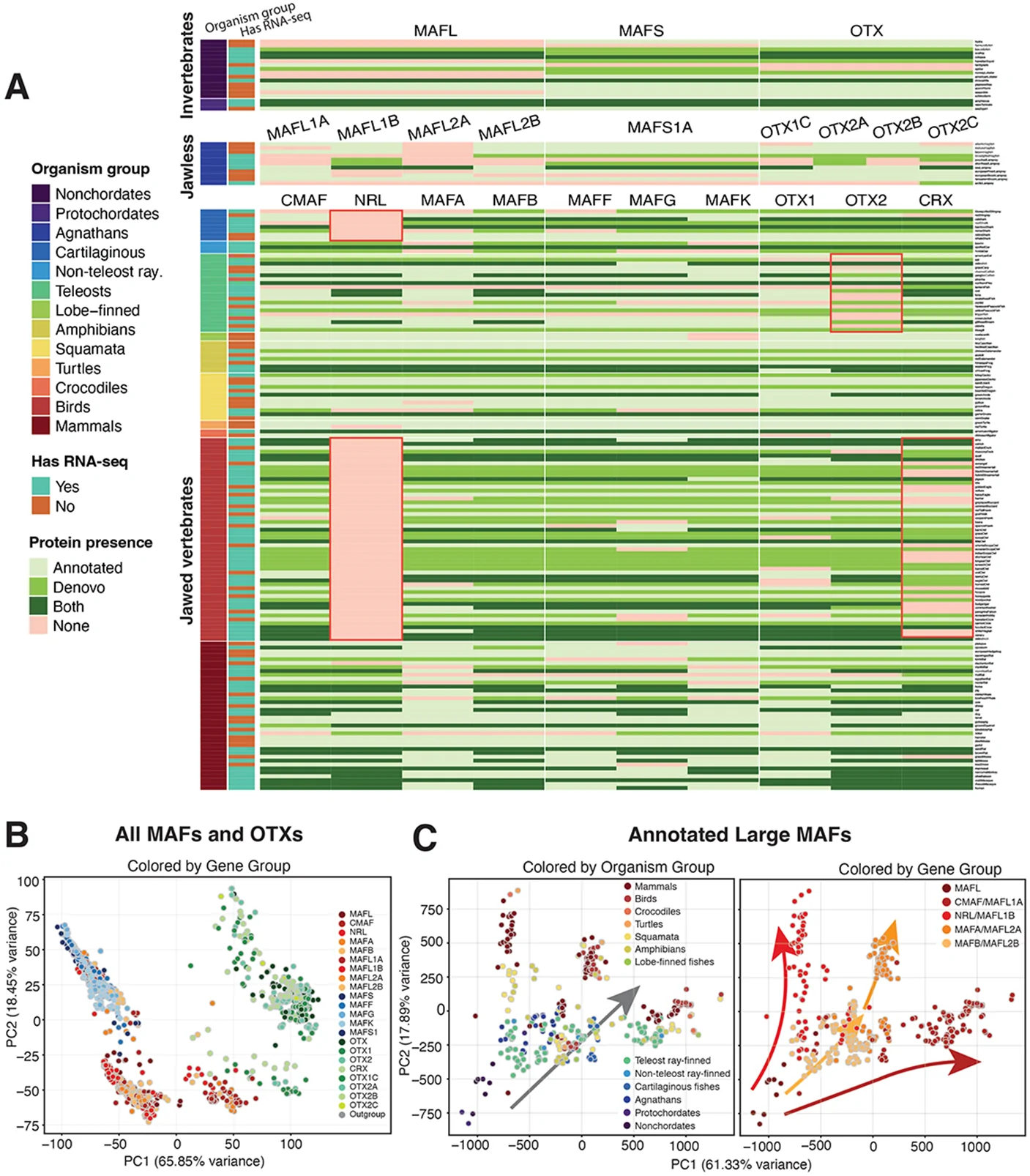
Presence and sequence similarity of MAF and OTX proteins across studied species. (A) Heatmap showing the presence of annotated and *de novo* predicted MAF and OTX proteins in organisms included in this study. Cell colors indicate protein presence: different shades of green depending on presence among the annotated, *de novo* predicted, or both datasets, while red indicates absence. Red borders denote notable group-specific missing homologs in annotated and/or *de novo*-predicted datasets. (B) Principal component analysis of ESMC embeddings for all MAF and OTX sequences (left) and large MAF proteins (middle and right). Graphs are colored by gene/family (left), organism group (middle) and gene group (right). Arrows indicate evolutionary trends.

**Figure 3.**
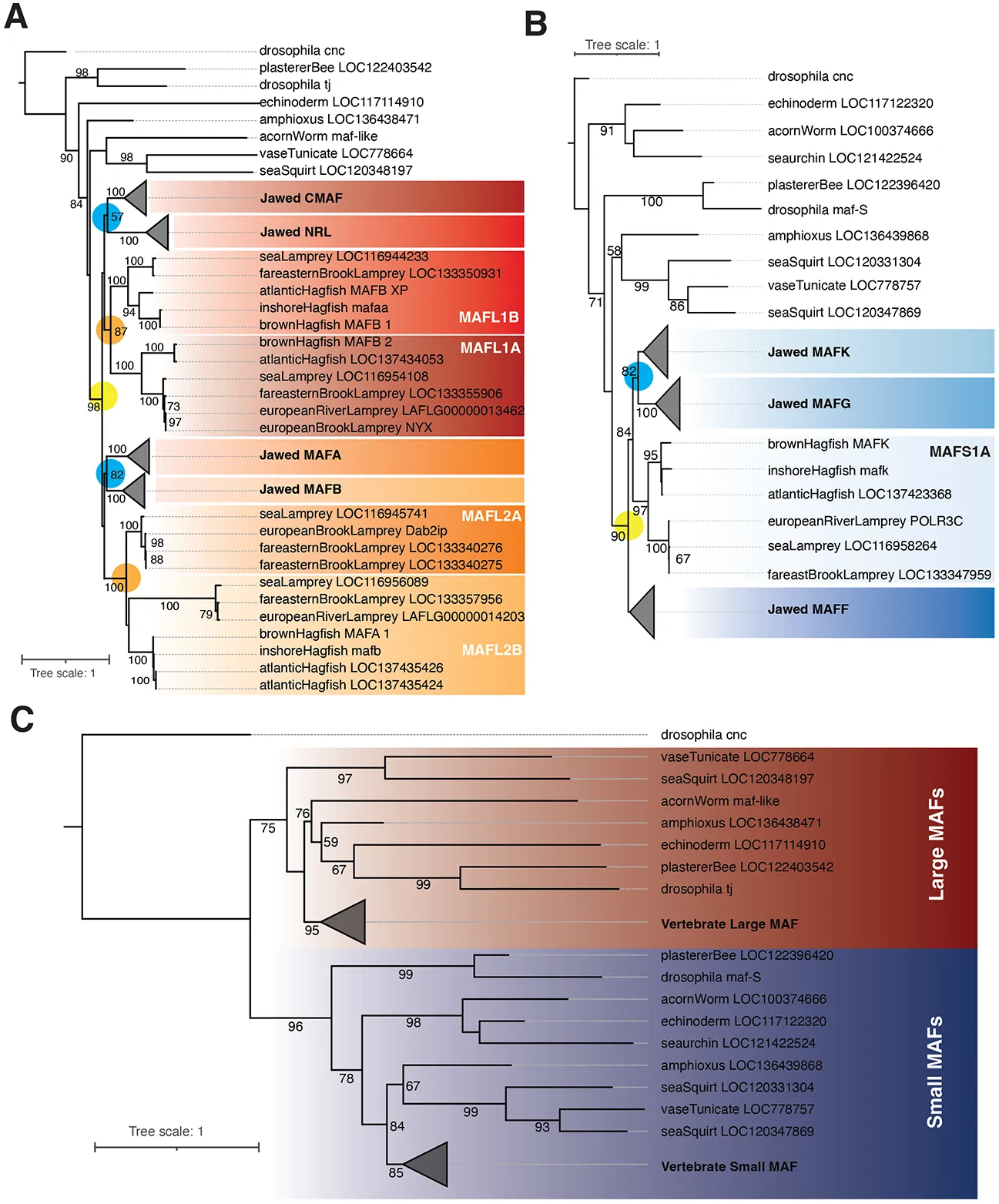
Large, Small, and combined MAF trees. (A) Phylogeny of large MAF proteins. The tree highlights four distinct clades in jawed vertebrates (cMAF, NRL, MAFA, and MAFB) and four distinct clades in jawless vertebrates. These clades appear to have arisen from a single common ancestral duplication event (yellow), followed by independent lineage-specific duplications in jawed vertebrates (blue) and jawless vertebrates (orange). (B) Phylogeny of small MAF proteins. The tree highlights three distinct clades in jawed vertebrates (MAFF, MAFG, and MAFK) and one distinct clade in jawless vertebrates. These clades appear to have arisen from a single common ancestral duplication event (yellow), followed by a jawed vertebrate-specific duplication (blue). C) Phylogeny of combined large and small MAF proteins. All trees were inferred from sequences representing 86 species and rooted using *Drosophila* “cap’n’collar”. Branches connecting the root to the outgroup and the ancestral large MAF were manually shortened for clarity. Bootstrap support values above 50% are shown on branches. Branch lengths are reported as substitutions per alignment site.

**Figure 4.**
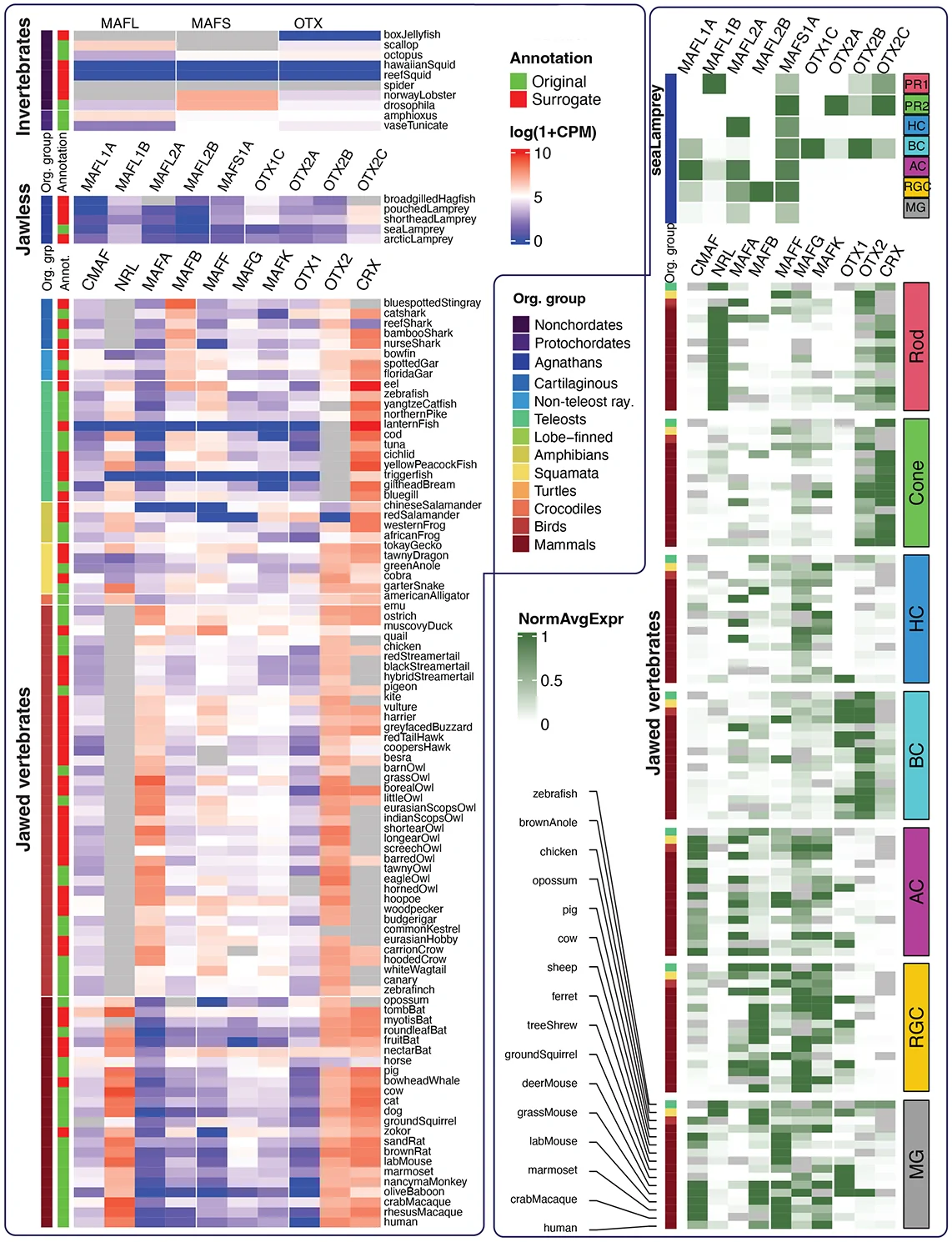
Evolution of large MAF and OTX expression in retina. (*Left*) Heatmap showing bulk RNA-seq expression of large MAF and OTX family genes across species. Rows represent species (grouped by clade, ordered and colored as in Fig. 1B&C); columns represent genes (grouped by family). The top panel shows invertebrate species, which possess a single ancestral copy each of large MAF, small MAF and OTX. The middle panel shows cyclostomes, which have four paralogs each in the large MAF and OTX families and a single small MAF paralog (as identified in this study). The bottom panel shows expression pattern of *cMAF*, *NRL*, *MAFA*, and *MAFB* (large MAF subfamily), *MAFF*, *MAFG*, and *MAFK* (small MAF subfamily) and *OTX1*, *OTX2*, and *CRX* (OTX family) in jawed vertebrates. Expression values are log-transformed counts per million (log-CPM) after TMM normalization, performed separately within each of the three species groups (invertebrates, cyclostomes, jawed vertebrates) using group-specific ortholog sets. *(Right*) Heatmap showing cell-type-specific expression of large MAF and OTX family genes from single-cell RNA-seq across species. Rows represent cell types; columns represent genes (grouped by family). Values are average log-normalized expression per cell type, scaled to [0,1] by dividing by the maximum value across all cell types within each dataset. The top panel shows data for the sea lamprey, which has four paralogs each in the large MAF and OTX families (as identified in this study). The bottom panel(s) show expression pattern large-, small MAF and *OTX* genes in jawed vertebrates.

**Figure 5.**
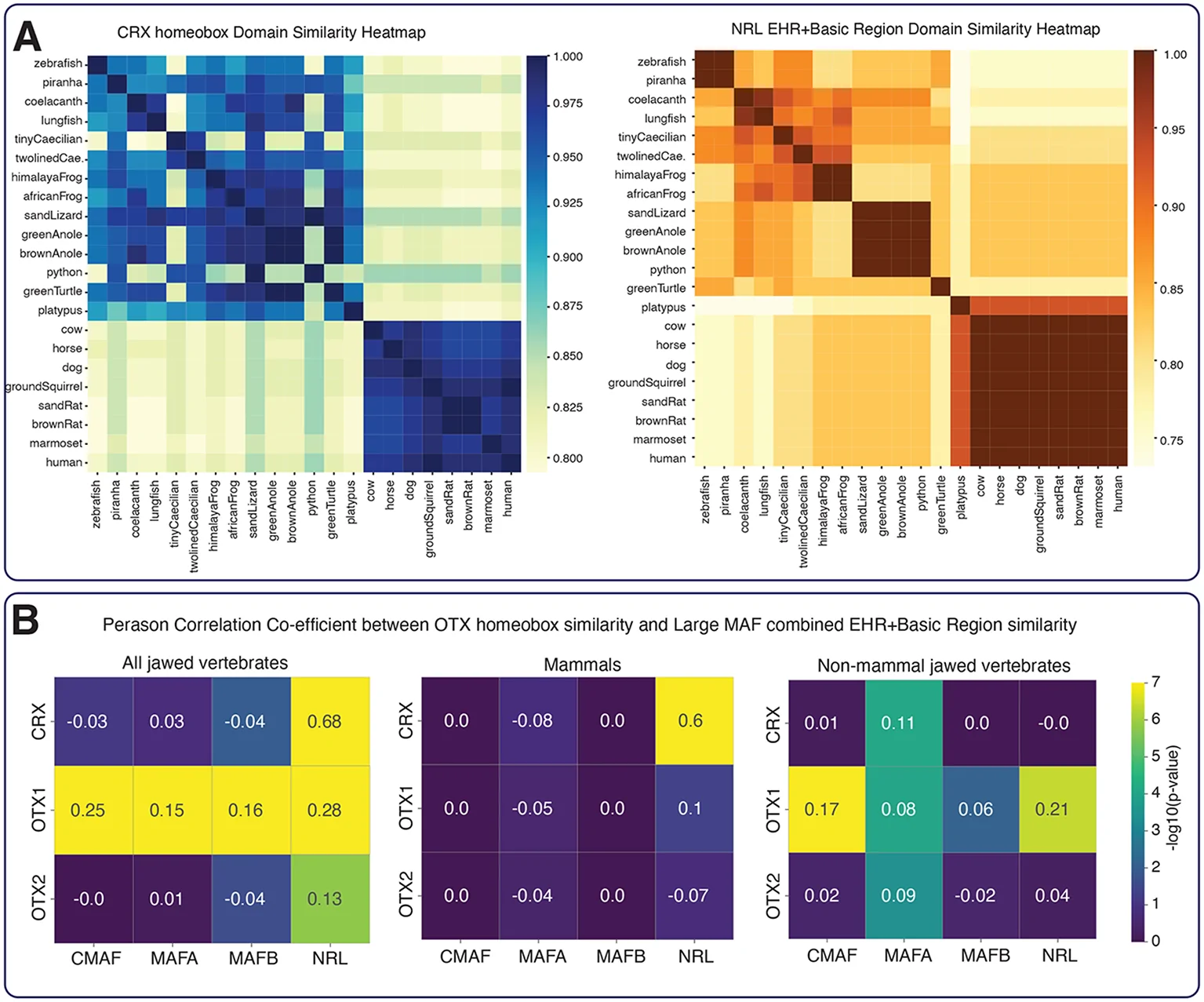
Co-evolution of vertebrate OTX and large MAF domains. (A) Representative pairwise sequence similarity heatmaps for the CRX homeodomain (left) and the NRL extended homology region (EHR) + basic region (right). The Pearson correlation coefficient (PCC) between these two similarity matrices (or their submatrices) corresponds to the CRX-NRL cell in each matrix shown in panel B. (B) Matrices of PCC values comparing sequence similarity of OTX homeobox domains with those of combined MAF EHR and basic domains. Results are shown for all jawed vertebrates (left), mammals (middle), and non-mammalian jawed vertebrates (right). Cell values represent PCCs, with color intensity indicating −log10(p-value) of the PCC (shared color scale across matrices).

**Figure 6.**
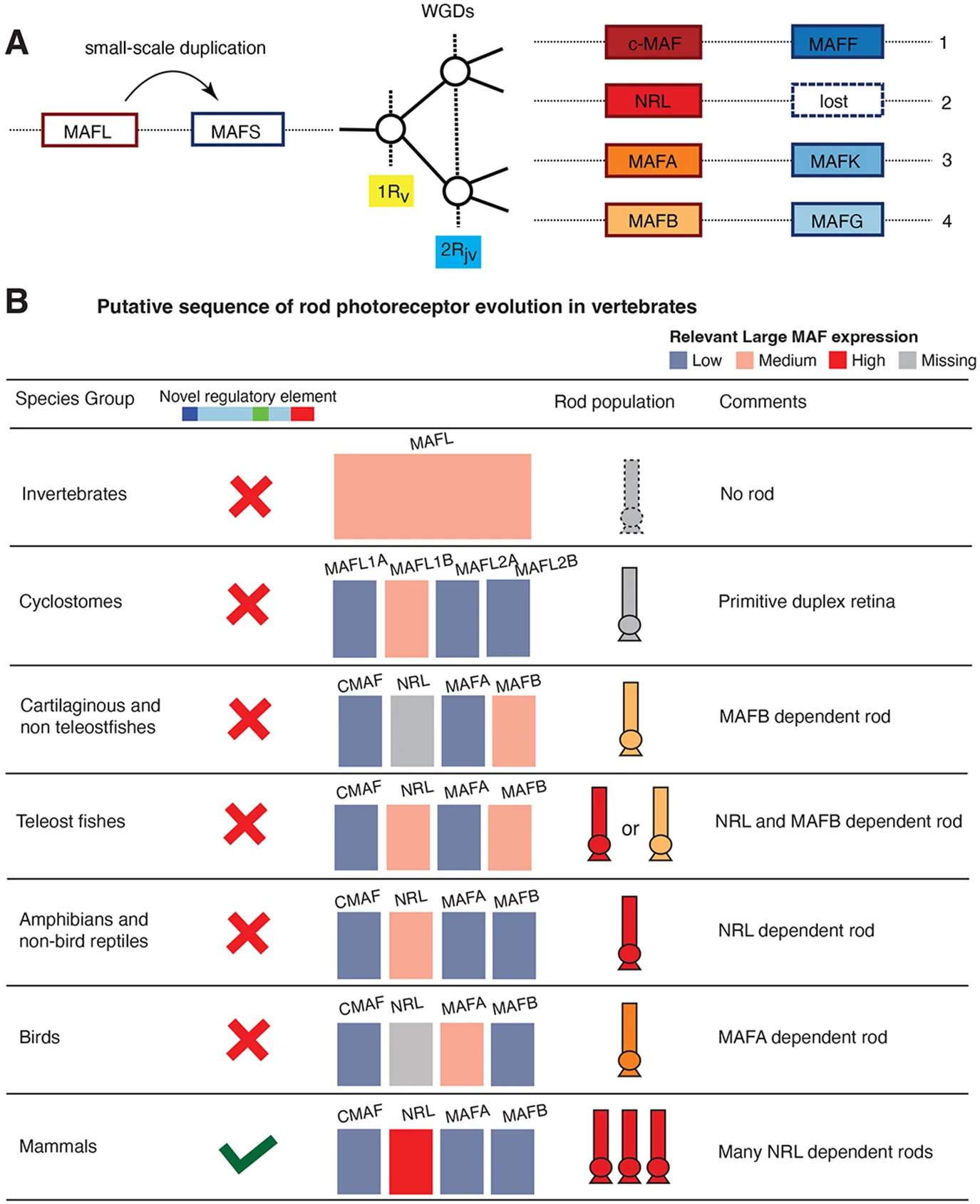
Evolution of MAF genes and proposed sequence of rod photoreceptor evolution in vertebrates. (A) Proposed simplified evolutionary model for the origin and divergence of MAF proteins (cyclostome-specific duplications omitted). An ancestral small-scale (segmental) duplication produced an ancestral small MAF (MAFS) from an ancestral large MAF (MAFL). These precursors then underwent two rounds of vertebrate WGDs (1R_v_ and 2R_jv_), yielding four large MAF paralogs (cMAF, NRL, MAFA, MAFB) and four small MAF paralogs (MAFF, MAFG, MAFK, and one lost). (B) The schematic table summarizes a hypothesized evolutionary model for the stepwise emergence and diversification of rod photoreceptors across major lineages, from invertebrates (outgroup) to mammals. Rows represent species groups arranged chronologically from top to bottom. Columns display: (1) Species Group, (2) Novel regulatory element: Red × indicates inferred absence; green ✓ (mammals only) indicates presence of a novel cis-regulatory element linked to NRL, (3) Relevant Large MAF expression: Colored bars depict relative expression levels of jawless vertebrate MAFs (*MAFL1A*/*MAFL1B*/*MAFL2A*/*MAFL2B*) or jawed-vertebrate MAFs (*CMAF*, *NRL*, *MAFA*, *MAFB*) large MAF-family transcription factors, (4) Rod population: Schematic rod icons illustrate type, color-coded dependency on specific large MAF protein, and relative abundance (e.g., crossed-out/absent, single primitive rod, yellow MAFB-dependent rod, red NRL-dependent rod, or multiple rods), (5) Comments: Interpretive summary of rod characteristics and proposed transcription-factor dependencies in each group. The model proposes that an ancestral MAFB-dependent rod mechanism in early jawed vertebrates transitioned to NRL-dependent rods in most tetrapods. Birds secondarily shifted to MAFA-dependent rods, whereas mammals acquired a novel regulatory element that drove highly elevated NRL expression and a substantial expansion of NRL-dependent rod photoreceptors.

## Data Availability

The raw and final analyzed data has been deposited in Gene Expression Omnibus (GEO) data repository with accession number: GSE332649. For review, GSE332649 data can be accessed using the token kfehgcigpjeznkb The data will be made freely available when the manuscript is accepted. Custom code used for data analysis is available in Zenodo repository (https://doi.org/10.5281/zenodo.18023142) and GitHub repository (https://github.com/NIH-NEI/maf_otx_evolution).
